# Cyclist Injury Severity in Spain: A Bayesian Analysis of Police Road Injury Data Focusing on Involved Vehicles and Route Environment

**DOI:** 10.3390/ijerph17010096

**Published:** 2019-12-21

**Authors:** Rachel Aldred, Susana García-Herrero, Esther Anaya, Sixto Herrera, Miguel Ángel Mariscal

**Affiliations:** 1 School of Architecture and Cities, Westminster University, London NW1 5LS, UK; R.Aldred@westminster.ac.uk; 2 Escuela Politécnica Superior, Universidad de Burgos, 09001 Burgos, Spain; mariscal@ubu.es; 3 Center for Environmental Policy, Imperial College London, London SW7 2AZ, UK; e.anaya14@imperial.ac.uk; 4 Meteorology Group, Applied Mathematics and Computer Science, Universidad de Cantabria, 39005 Santander, Spain; sixto.herrera@unican.es

**Keywords:** cycling, road safety, injured cyclist, Bayesian network, data mining

## Abstract

This study analyses factors associated with cyclist injury severity, focusing on vehicle type, route environment, and interactions between them. Data analysed was collected by Spanish police during 2016 and includes records relating to 12,318 drivers and cyclist involving in collisions with at least one injured cyclist, of whom 7230 were injured cyclists. Bayesian methods were used to model relationships between cyclist injury severity and circumstances related to the crash, with the outcome variable being whether a cyclist was killed or seriously injured (KSI) rather than slightly injured. Factors in the model included those relating to the injured cyclist, the route environment, and involved motorists. Injury severity among cyclists was likely to be higher where an Heavy Goods Vehicle (HGV) was involved, and certain route conditions (bicycle infrastructure, 30 kph zones, and urban zones) were associated with lower injury severity. Interactions exist between the two: collisions involving large vehicles in lower-risk environments are less likely to lead to KSIs than collisions involving large vehicles in higher-risk environments. Finally, motorists involved in a collision were more likely than the injured cyclists to have committed an error or infraction. The study supports the creation of infrastructure that separates cyclists from motor traffic. Also, action needs to be taken to address motorist behaviour, given the imbalance between responsibility and risk.

## 1. Introduction

Cyclists are considered ‘vulnerable road users’ because, like pedestrians, they are at relatively high risk of serious injury compared to drivers of motor vehicles. However, recent research highlights the overall population health benefits that result from cycling implying the need to increase active travel as well as to make it safer [1].

Much previous research on cyclist injury severity has examined cyclist characteristics, often focusing on helmet wearing and head injuries [2]. Another group of studies examined (mis)behaviours including use of alcohol or drugs [3]. Other research has targeted demographic correlates of injury risk, finding older people to be more vulnerable to severe injury [4]. Another strand of work concentrated on route conditions, from permanent fixed infrastructure to temporary conditions such as weather or light levels [5,6,7,8]. In some studies, bicycle infrastructure has been found to reduce injury severity, as have street characteristics such as lower speed limits, and secondary or tertiary roads compared to primary roads. There is relatively little work studying factors relating to drivers, although some work did examine vehicle types, finding that larger vehicles—particularly lorries—are associated with higher injury severity [9].

Most research effort focused on Anglophone countries, or on high-cycling contexts such as Denmark. Here the study setting is Spain, a European country with generally low cycling rates. The first priority listed in the Spanish Road Safety Strategy 2011–2020 [10] is “to protect the most vulnerable users”. One of the Strategy’s 13 goals is to achieve 1,000,000 more cyclists without an increase in what is described as the ‘cyclist death rate’ at baseline (2009). Yet while the number of cycling deaths remained stable from 2007 to 2016 (66 ± 12), Spain saw a 60% increase in hospitalised cyclists and a 215% increase in cyclist injuries not requiring a hospital stay [11]. For all injured casualties, in the same period of time (from 2007 to 2016), the percentage of cyclists almost doubled, from 4% to 7% [12]. Alongside this substantial growth in injuries, growth in cycling remains uneven across Spain. While some cities, such as Seville, have seen major growth from a low base [13], others, such as Madrid, continue to have very low cycling levels. Given unevenness in take-up alongside growing injury numbers, there is debate about how best to increase cycling uptake and cycle safety.

Unfortunately, Spain no longer conducts a regular national travel survey. The 2006/2007 Movilia survey is the most recently available data, ten years earlier than the injury data we are analysing. Hence, we are unable to assess injury risk in relation to exposure. Instead, the paper aims to explore whether a diverse range of factors are associated with cyclist injury severity in the Spanish context. In this way it contributes to discussion about how to reduce the risk of severe injury for cyclists involved in collisions.

This study investigates the following questions:

(i) What route environment, vehicle, and rider/driver-related factors are associated with elevated cyclist KSI risk (Risk of being killed or seriously injured, if involved in an injury collision recorded by the police)?

(ii) What interactions can be identified between these factors?

## 2. Methods 

### 2.1. Approach

Authors have investigated and studied the causes of road traffic collisions using diverse methods and techniques [14]. Traditionally, classical statistical methods such as regression models, ordered probit models, and decision trees have been used to predict the severity of traffic collisions and to determine contributing factors. Moreover, artificial intelligence techniques like genetic algorithms, artificial neural networks, principal component analysis and fuzzy logic have been widely used in injury prediction models [15].

Recently, the number of Studies using Bayesian networks in safety context is rising as this method provides reliable inferences regarding safety issues [16,17,18,19,20,21,22,23]. This includes evaluating the severity of traffic collisions, analysing their causes and/or predicting the probability of fatal and serious injuries. Previous research efforts demonstrated that Bayesian networks predict collision severity better than traditional methods such as regression models [24,25]. 

This study proposes a Bayesian network model in which the outcome variable is ‘KSI’ which represents the injury severity (killed or serious injured versus slightly injured) experienced by an injured cyclist in a collision. By using vehicle-level data (both cyclists and other involved vehicles) the study examines the extent to which factors related to injured cyclists and other parties, alongside route environment factors, are associated with a cyclist being killed or seriously injured in a collision.

### 2.2. Variables

Data used in this study have been collected from the 2016 traffic collision database provided by the Directorate General of Traffic, which is responsible for managing the "National Registry of Victims of Traffic Accidents” in Spain [26]. Variables used in this study are fully described in Table 1 and relate to cyclist/motorist behaviour and route environment conditions. The rest of this subsection provides context for international readers about three key types of infrastructure/zonal characteristics related to urban zone or not, presence of bicycle infrastructure, and traffic calming. The dataset classifies a location as a ‘calle’ (“street”) or not. ‘Calle’ here is translated as ‘urban zone’, with other locations comprised of inter-urban zones or urban highways which are also defined as inter-urban.

Spanish traffic regulations [27] refer to several types of bicycle infrastructure; “cycle lane” (on-road cycle path), “protected cycle lane” (on-road cycle path with some kind of physical protection), “sidewalk cycle path” (delimitated cycle path located on pedestrian spaces), “cycle track” (completely separated from the rest of the traffic) and “cycle path” (separated from traffic, shared with pedestrians, and within green spaces). The variable ‘30-zone’ might be best defined as ‘traffic calmed’, including streets where speed limits are lower than 30 kph or where motor traffic is excluded or restricted. Within urban areas generally, the default national speed limit is 50 kph. However, municipalities can install 30 kph and 20 kph zones and some, including Barcelona and Madrid, have implemented 30 kph limits across much of the city.

### 2.3. Model

This section describe the principle of the Bayesian network model which is implemented in MATLAB software (Matlab 2014b). In the proposed Bayesian Network model, in which the outcome variable is the cyclist severity injury (KSI), the Bayes Classifier (BC) minimizes the probability of misclassification by solving the following optimization problem:

(1) $$argmax_{KSI}\left[P\left(KSI|\left\{X_{1},\ldots ,X_{n}\right\}\right)\right],$$

Discrete Bayesian Networks (BN) are probabilistic graphical models to learn the joint probability distribution (JPD) of a multivariate problem involving multinomial variables (22). The model is based on a directed acyclic graph that expresses the direct/conditional dependencies/independencies between the variables and simplifies the learning of the JPD, based on the factorization associated to the independencies given by the directed acyclic graph, DAG (Equation 1), and the interpretability of the resulting model. The equation 1 represents the Joint Probability Function of the Bayesian Network. Where $\left\{x_{1},\ldots ,x_{n}\right\}$ are the variables considered in the model and $\pi_{i}$ are the set of parents of the variable $x_{i}$ given by the DAG.

(2) $$p\left(x_{1},x_{2},x_{3},\ldots ,x_{n}\right)=\prod_{i=1}^{n}p\left(x_{i}|\pi_{i}\right),$$

As a result, the learning is divided in two phases: structural and parametric. First, the DAG is obtained by applying the greedy learning algorithm proposed by Buntine [28]. This is a score-based algorithm that tries to obtain the DAG corresponding to the lowest Bayesian Information Criterium (BIC) which is a measure of the goodness of fit of a Bayesian model based on the likelihood function that penalizes the complexity of the model to avoid overfitting (See Schwarz [29] and Wit [30] for detailed explanation of the score definition). To this aim, for each step the algorithm evaluates all the possible links between the variables introducing the DAG with lowest BIC that best represent the independencies of the data. Please note that we have not included a minimum improvement threshold to add new links to the graph due to the penalty term of the BIC, limiting the inclusion of new links. Other algorithms introduce a pre-order of the variables (K2-algorithm, [31]) limiting the possible parents of a particular variables in order to reduce the computational costs but introducing a dependence on the order established, so we decided discard this option. Secondly, the parameters given by the DAG are obtained by maximum likelihood as the ones that better explain the observed data. Note that the DAG doesn’t reflect causality but the statistical dependences between the variables, and from a mathematical point of view an equivalent factorization can be obtained keeping the non-directed graph and the v-structures relating three variables (e.g., Age → Gender ← Helmet), but it is not necessary to maintain the direction of the links between variables [31].

Based on the resulting JPD and DAG, new knowledge for one or several variables of the model (evidence) can be easily propagated to the rest of the BN obtaining the new probabilities of the rest of variables included in the model (inference).

(3) $${\mathrm{JPD}}_{\mathrm{BN}}=p\left(\mathrm{KSI},X_{1},\ldots ,X_{n}\right),$$

Finally, the resulting JPD of both, factors and target variable (KSI), allows us to define a natural BC by establishing a threshold above/below which the occurrence/absence of KSI is identified.

According to the objectives of the study, two experiments were defined. First, a 10-fold cross-validation experiment was developed to obtain the skill and generalization capabilities of our Bayesian Network, and to identify possible biases. To this aim a random partition of the database in 10 subsets was defined. For each subset 90% of data used for training and the remaining used for predicting. As a result, a prediction of the full sample is obtained by joining the ten subsets. Several parameters have been considered to evaluate the resulting model. The Area Under the Receiver Operating Characteristic Curve (AUC, [32]) was used to evaluate the skill for both each fold and the full series obtained by joining the ten predictions. This measure is based on the ROC Curve, that plots the Hit Rate versus the False Alarm Ratio as the probability threshold varies, obtained by integrating the curve. The score varies between 0 (opposite predictor) and 1 (perfect predictor), being the 0.5 equivalent to a random predictor system. The result of the AUC in the present study was between 0.91 and 0.95.

As the AUC can be biased to one of the categories, mainly when there is an unbalance in the sample to a state of the variable, the sensitivity and specificity have been defined as follows:

(4) Sensitivity=TP/P∧Specificity=TN/N,

where TP/TN stands for the number of predicted True Positives/Negatives, and P/N the number of observed Positives/Negatives, respectively. Furthermore, the accuracy index was defined as:

(5) $$\mathrm{Accuracyindex}=\frac{\mathrm{TP}+\mathrm{TN}}{P+N},$$

The results of the Sensitivity and Specificity for KSI in the present study were 0.60 and 0.99 respectively, and the accuracy index was 0.95. 

Secondly, taking advantage of the properties of the BNs, a sensitivity analysis was proposed by evaluating how the KSI’s probability changes when different factors are evidenced.

Note that only events without missing data in both the factors and target variable, which corresponds to the 99.7% of the sample size, have been considered. This approach lets us to consider a unique model for the sensitivity analysis, removing bias related with the sample, avoiding problems in the model adjustment, and prevents the introduction of noise in the results due to any filling gaps procedure or the availability of different variables for each event. In addition, once the model has been evaluated and its predictability tested, 100% of the database has been considered to train the model used for the sensitivity analysis.

Many programs have been developed to efficiently train Bayesian Networks, such as Netica Software, Hugin Investigator, Genie, Matlab, R or Microsoft with MSBNx sotfware. For our study, we used the Bayesnet toolbox for Matlab (Matlab 2014b).

## 3. Results

### 3.1. Descriptive Statistics

In 2016 there were 102,362 injury collisions on Spanish roads, involving 179,295 vehicles and 174,679 drivers or riders, of whom 12,318 were involved in a cyclist collision with at least one injured cyclist. This included 7488 cyclists involved in these collisions, of whom 66 were killed and 711 seriously injured. A collision that injures a cyclist is unlikely to injure a motorist: of 4830 drivers or motorcyclists involved in collisions with cyclists, 4626 (95.8%) were uninjured; 184 (3.8%) sustained a slight injury, with 17 (0.4%) seriously injured and 3 (0.1%) killed (see Table 1). 

As presented in the last row in Table 2, of the 7488 cyclists involved, 4880 (65.2%) were involved in collisions with motor vehicles. The other 2608 cyclists were involved in falls or in collisions involving other cyclists (the injury data contained records relating to 258 cyclists who were not injured but were involved in a collision that injured other cyclists) (see the last variable in Table 2). 

Here we focus on factors related to motorised vehicle involvement, also presenting analysis related to the 2608 (34.8%) of cyclists injured in falls or collisions involving other non-motorised users. Within non-motorized incidents the number of pedestrian-cyclist collisions is unknown, and in this case a pedestrian-cyclist collision is considered as cycle-only collision. Of the motor vehicles involved in cycle collisions, 4262 (88.2%) were cars; 313 (6.5%) were motorcycles; 155 (3.2%) were HGVs; 53 (1.1%) buses; and 47 (1.0%) other types of vehicle.

Table 1 shows the distribution of our modelled variables in relation to cyclists and to the motorists involved in these injury cyclist collisions. Key descriptive findings highlight the distributions of different crash types. While 7% of cyclists were involved in an incident taking place on cycling infrastructure, only 0.2% (10) of motor vehicles collided with a cyclist on cycling infrastructure. In other words, cycle infrastructure seems to sharply reduce the likelihood of collision with a motor vehicle, with non-motorised falls/collisions being more typical. Similar but less striking findings are true for urban zones (where non-motorised crashes are more typical than in inter-urban zones), but not for 30 kph zones. Over three-quarters of collisions took place in clear, dry weather, without any surface related issues, with the same true for visibility, although with a very high number of missing values. 

Cyclists and motorists have different age profiles; specifically, children are unsurprisingly almost absent among the latter category. By contrast, those aged under 18 made up 10.8% (806) of injured cyclists. Men dominated among both cyclists and motorists, but even more so among cyclists (83.1% vs. 73.4%). Just under half of the involved cyclists (44.9%) were recorded as definitely having worn a helmet, albeit with a high level of missing data. 

Finally, the data record the prevalence of infractions and errors attributed both to cyclists and involved motorists. Motorists, while unlikely to have sustained an injury, are relatively more likely to have committed an infraction or error, compared to cyclists. For other drivers and riders, 32.0% (1544) were recorded as having committed an infringement of some type, while for cyclists the figure was 14.1% (1057). For errors, the respective figures are 22.4% (1084) and 12.8% (960).

### 3.2. Factors Associated with High Risk

**Figure 1:**
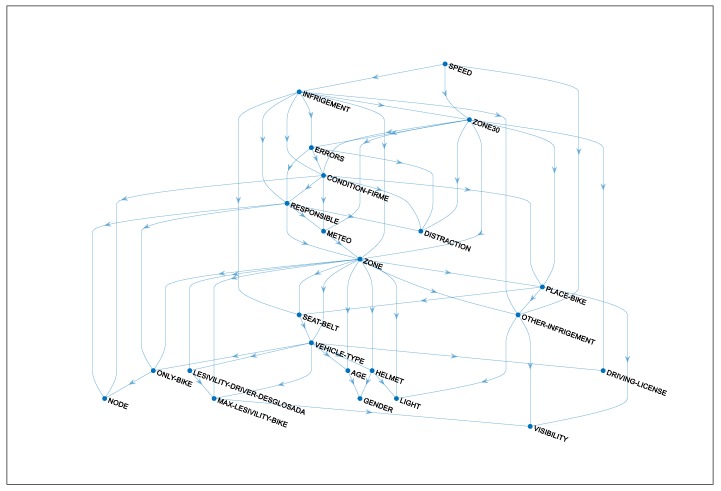
Directed acyclic graph obtained.

Table 3 highlights factors associated with high KSI risk for cyclist, based on our Bayesian network model (see the directed acyclic graph in Figure 1). The table shows the probabilities of a cyclist being killed or seriously injured by effect of each variable. 

The involvement of an HGV is associated with an elevated risk of death or serious injury (23.9%, compared to 13.3% for buses and 10.1% for cars) as is the involvement of ‘other’ vehicles. Conversely, collisions involving motorcycles are associated with lower risk of death or serious injury to the cyclist (8.1%). Perhaps surprisingly, non-motorised collisions are associated with a higher KSI risk (12.0%) than collisions involving cars (10.1%) or motorcycles (8.1%). However, this overstates the severity of the risks related to these collisions, as few deaths (as opposed to serious injuries) occur in non-motorised incidents. Of 541 KSI cyclist incidents involving motor vehicles, 71 (13.1%) were deaths, while of 320 KSI cyclist incidents not involving motor vehicles, only 11 (3.4%) were deaths.

Other notable findings relate to the location. A location with bicycle infrastructure is associated with a somewhat lower risk of KSI compared to one without (8.8% vs. 12.2%), while an urban zone has a lower risk of KSI than an inter-urban zone (7.9% vs. 19.0%), as does a 30 zone (8.3% vs. 11.9%). Other factors had little relationship to injury severity, apart from poor visibility.

Table 4 illustrates the probability of the cyclist injury severity risk associated with factors specific to the motorist or the cyclist involved in the incident. The Bayesian network inference was generated after turning ‘vehicle type’ into two discrete states (bicycles and other vehicles). Motorists aged between 40–60 and those under 18 had an elevated risk of seriously injuring a cyclist, while middle aged adults had a somewhat elevated risk of being severely injured. No gender differences were found, nor were there much differences in behaviour terms, such as whether the driver was wearing a seatbelt, or carrying the correct drivers’ licence. 

The motorist not using a helmet (mostly referring to motorcyclists) had more probability of seriously injuring a cyclist, 16.1% versus 13.6%. Perhaps surprisingly, helmet use among cyclists was associated with higher risk of severe injury (14.2%, vs. 10.8%). Infringements and responsibilities in the incident did not appear to influence the injury severity of the cyclist, but as demonstrated above, the level of culpability for drivers is higher than for cyclists.

### 3.3. Interactions between Vehicle Type and Route Environment

Table 4 has shown that certain types of route environment (bicycle infrastructure, 30 kph/traffic calmed zone, and urban zones) are associated with lower risk of serious injury for people cycling, while larger vehicles (particularly HGVs) are associated with elevated KSI risk. This section provides data on interactions between vehicle type and injury severity. For instance, traffic calmed areas reduce KSI risk in general, but do they specifically mitigate risks for crashes involving the most dangerous vehicles, such as HGVs?

Before presenting the analysis, Table 5 shows the likelihood of (i) an injured cyclist and (ii) an involved motor vehicle being present in different types of location. Except for motorcycles (who are not allowed to use bicycle infrastructure, but nevertheless may sometimes do so) few motor vehicles were involved in collisions with cyclists on bicycle infrastructure. Urban zone-based collisions dominated across vehicle groups except HGVs, where slightly more than half of all collisions took place in non-urban zones. Collisions with HGVs were particularly unlikely to happen on both dedicated cycle infrastructure and on 30 zones.

Table 6 presents the KSI risk by zone type (urban/non-urban and 30-zone/others) based on the involvement of different motor vehicles. A gradient can be seen both for vehicle type (if ordered by weight: Motorcycles, Cars and then Trucks and buses) and for location type. For instance, the KSI risk associated with truck involvement is 14.1% for urban zones; higher than for all other road user types except ‘others’, but lower than the KSI risk associated with truck involvement in inter-urban zones (32.5%).

As there were few cases of collisions with motor vehicles on bicycle infrastructure, they could not be split up by type of motor vehicle involved. Instead, Table 7 separates collisions involving motor vehicles or not and compares KSI risk by presence of cycle infrastructure. In both cases, presence of bicycle infrastructure reduces injury severity.

## 4. Discussion

This study found a higher risk for cyclists in Spain of being killed or seriously injured where HGVs are involved in a collision, compared to other vehicles, which is consistent with previous studies that used national-level data [33]. Motorists involved in collisions that injure cyclists are highly unlikely to be killed or seriously injured; in 95.8% of cases they are uninjured. However, according to the police, involved motorists are around twice as likely as the injured cyclists who have committed an infraction or made an error which is consistent with the findings of Bíl et al. for the Czech Republic [34]. The study did not find a protective effect associated with helmets but an increase of risk. Most studies have reported a protective effect of helmet wearing in relation to head injuries and fatality [35]; however, other studies have found helmet wearing may increase the risk of other type of injuries [36].

The research did find a reduction in KSI risk associated with three infrastructure categories: bicycle infrastructure, urban zones (excluding major roads within these), and 30 kph or less zones (reduced speed limit, pedestrianised, and/or residential areas). Few collisions involving motor vehicles happened at locations with bicycle infrastructure, and where bicycle infrastructure was present both collisions involving motor vehicles and those not involving motor vehicles were less likely to be serious. Sensitivity analysis focused on urban and 30 kph zones (due to only 10 cases of motor vehicle collisions on bicycle infrastructure) and found that this reduction in KSI risk held for all vehicle types.

The results in relation to each of the categories found to be protective are aligned with the findings in literature. Reynolds et al.’s review [8] documented the protective effects of bicycle infrastructure. No review comparing cycling injury risk in urban vs. rural roads has been found, but our results are consistent with the studies that used national databases [5]. Cleland et al reported that the introduction of 20 mph (approx. 30 kph) zones would decrease cyclist-involved collisions [37]. 

## 5. Limitations and Generalisability 

It is well known that police injury data do not capture all injuries, and in particular slight injuries are under-represented. Under-representation of cyclist-involved collisions have been evidenced over-time and at international level [38,39] and it is an intrinsic limitation of this study due to the use of police data [40]. Police definitions of ‘serious injury’ also cover quite diverse levels of injury. 

As not all regions in Spain used the same road collision data collection system during 2014 and 2015, this study has been carried out only with data for 2016 that contains few cases of deaths. Therefore, we were unable to define the target variable in four states (no injury, minor injury, serious injury or death). Instead, we used the KSI variable by combing serious and fatal injuries. This makes the study results consistent as the resulting model has a favourable accuracy index (0.95).

Some findings may not be specific to the Spanish context. For instance, the low KSI risk following a motorcycle collision may not be transferable to other countries with different motorcycle usage: in Spain, 10% of registered motor vehicles are motorcycles [12].

The counter-intuitive finding for helmets may be related to Spanish helmet laws as helmets are compulsory on non-urban roads, where the risk is higher. The probability of an injured cyclist having used a helmet in inter-urban zones calculated with Bayesian network inference was 81.6%, compared to only 28.1% on urban areas. This is likely to be in turn related to different types of cyclist and cycling, not controlled for in this analysis. Hence the results showing a higher KSI risk for helmet-wearing cyclists may not transfer to other contexts.

## 6. Conclusions 

The study suggests that separating cyclists from motorised traffic (as via bicycle paths) and/or reducing levels and speeds of motor traffic (as 30 zones, which include pedestrianised and residential streets, aim to do) can help reduce cyclist injury severity. For example, in collisions involving motor vehicles, the presence of cycle infrastructure reduces the probability of KSI risk from 11.8% to 8.6%. This happens partly by reducing the likelihood of collisions with motor vehicles, particularly HGVs: in Spain, HGVs are often restricted in pedestrian and residential areas. However, where interactions with other vehicles do appear (which might happen, for instance, where pedestrianized areas allow timed loading by larger vehicles) the risk of serious injury to the cyclist is reduced (9.6% KSI risk in collision not involving motor vehicles versus 8.6% KSI risk in collision with other vehicles).

The study thus supports the creation of bicycle infrastructure and/or traffic-calmed/30kph zones in urban areas. It highlights the relatively high risk associated with major roads, often outside urban zones (19% of KSI in inter-urban zones versus 7.9% in urban areas). Despite eight of ten injured cyclists in inter-urban zones having worn a helmet, these zones are associated with high risk of severe injury, perhaps partly due to risky overtaking manoeuvres on rural roads, with Spain’s legal minimum passing distance of 1.5 m being insufficient at high speeds [41] or in poorly maintained roads. Implementation of cycle infrastructure on these roads (rare in Spain, where bicycle infrastructure has mostly been built in urban areas and most of the country lacks supra-local cycle network planning) is further recommended. 

Finally, the study finds high rates of infractions (19% of cases versus 7.9%) and errors (22.4% versus 12.8%) committed by motorists involved by comparison to involved cyclists. Although those operating motor vehicles must pass a test and undergo licensing, they seem more likely than cyclists to be culpable in collisions that injure cyclists. While not associated with higher injury severity, such poor driving may contribute to the higher overall collision risk that cyclists experience, per km, on the roads, compared with motorists. Hence as well as infrastructure there is a role for driver education and enforcement focused around behaviour towards cyclists.

## Figures and Tables

**Table 1:** Factors related to cyclists and to other road users in injured cyclist collisions.

Variables	Nº Total Cases	% Cases	Nº Cases Cyclists	% Cases Cyclists	Nº Cases Non Cyclists	% Cases Non Cyclists	Comments
**Vehicle involved in an incident in which a cyclist was injured**							
**Bicycle**	7488	60.8%	7488	100.0%	0	0.0%	**Bicycle**
**Car**	4262	34.6%	0	0.0%	4262	88.2%	**Car, van, SUV**
**Motorcycle**	313	2.5%	0	0.0%	313	6.5%	**Moped, motorcycle (125 cc)**
**Lorry/truck**	155	1.3%	0	0.0%	155	3.2%	**Truck, articulated truck, articulated vehicle**
**Bus**	53	0.4%	0	0.0%	53	1.1%	**Minibus (up to 17 passengers), bus, articulated bus**
**Others**	47	0.4%	0	0.0%	47	1.0%	**Other vehicles**
**Bicycle infrastructure present?**							
**Yes**	537	4.4%	527	7.0%	10	0.2%	**Footway bicycle lane, bicycle lane, protected bicycle lane, “Pista-bici” (cycle track shared with pedestrians)**
**No**	5804	47.1%	3273	43.7%	2531	52.4%	
**Unknown**	5977	48.5%	3688	49.3%	2289	47.4%	
**Zone**							
**Urban**	8740	71.0%	5138	68.6%	3602	74.6%	**Street**
**Inter-urban or urban highway**	3572	29.0%	2344	31.3%	1228	25.4%	**Highway/motorway, road, secondary road**
**Traffic calming (30 kph or less)**							
**Yes**	1214	9.9%	702	9.4%	512	10.6%	**Residential, pedestrian areas, zone limited to 30 kph, any other area under speed reduction regulations**
**Others**	6329	51.4%	3843	51.3%	2486	51.5%	**Peri-urban area, ring roads**
**Unknown**	4769	38.7%	2937	39.2%	1832	37.9%	
**Intersection**							
**Yes**	5924	48.1%	3205	42.8%	2719	56.3%	**At a junction**
**No**	6388	51.9%	4277	57.1%	2111	43.7%	**Not at a junction**
**Weather conditions**							
**Clear**	9329	75.7%	5606	74.9%	3723	77.1%	**Clear day, sunny, not cloudy**
**Other**	759	6.2%	450	6.0%	309	6.4%	**Cloudy, light rain, heavy rain, hailing, snowing**
**Unknown**	2230	18.1%	1432	19.1%	798	16.5%	
**Surface**							
**Good**	10,413	84.5%	6246	83.4%	4167	86.3%	**Dry and clean**
**Others**	867	7.0%	596	8.0%	271	5.6%	**Sandy or gravel, wet, waterlogged or flooded, icy, snowy, oily, other**
**Unknown**	1038	8.4%	646	8.6%	392	8.1%	
**Light**							
**Good**	10,072	81.8%	6189	82.7%	3883	80.4%	**Natural daylight**
**Others**	2240	18.2%	1293	17.3%	947	19.6%	**Sunrise or sunset, night-time, without natural light, with artificial light or without artificial light or any light**
**Unknown**	6	0.0%	6	0.1%	0	0.0%	
**Visibility**							
**Good**	3619	29.4%	2245	30.0%	1374	28.4%	**Good visibility**
**Others**	861	7.0%	506	6.8%	355	7.3%	**Buildings, facilities or elements on the road, atmospheric factors, blinded by sun, artificial lighting or headlights of another vehicle, works, vegetation or trees, decorative elements, other objects on the road, panels and advertising, others**
**Unknown**	7838	63.6%	4737	63.3%	3101	64.2%	
**Age**							
**<18**	818	6.6%	806	10.8%	12	0.2%	
**18–25**	1234	10.0%	849	11.3%	385	8.0%	
**25–40**	8357	67.8%	4800	64.1%	3557	73.6%	
**40–60**	1648	13.4%	879	11.7%	769	15.9%	
**>60**	261	2.1%	154	2.1%	107	2.2%	
**Gender**							
**Men**	9766	79.3%	6223	83.1%	3543	73.4%	
**Women**	2468	20.0%	1223	16.3%	1245	25.8%	
**Unknown**	84	0.7%	42	0.6%	42	0.9%	
**Driving licence**							
**Yes**	2682	21.8%	0	0.0%	2682	55.5%	**Correct driving licence**
**No**	194	1.6%	0	0.0%	194	4.0%	**Not carrying a valid licence with them. Redeemed, inappropriate, timed out, cancelled or suspended, never had a licence, exhausted all licence points (in Spain there is a penalty points system for drivers)**
**Unknown**	9442	76.7%	7488	100.0%	1954	40.5%	
**Seat-Belt**							
**Yes**	2935	23.8%	0	0.0%	2935	60.8%	**Seat-belt fastened**
**No**	230	1.9%	0	0.0%	230	4.8%	**Seat-belt not fastened**
**Unknown or N/A**	9153	74.3%	7488	100.0%	1665	34.5%	
**Helmet**							
**Yes**	3627	29.4%	3363	44.9%	264	5.5%	**Wearing a helmet or it was apparently expelled**
**No**	2148	17.4%	2134	28.5%	14	0.3%	**Not wearing a helmet**
**Unknown**	6543	53.1%	1991	26.6%	4552	94.2%	
**Infringement**							
**No infraction**	4402	35.7%	3119	41.7%	1283	26.6%	**No presumed infraction**
**Yes**	2601	21.1%	1057	14.1%	1544	32.0%	**Not obeying the STOP sign, failing to "give away", not obeying the traffic light, not obeying generic priority rule, not respecting a signalised pedestrian crossing, not obeying the indications of an agent, not obeying other priority signs of way, partially invading the opposite direction, zigzagging, turning or changing direction illicitly, illicit driving in reverse gear, stopping without a due cause, not keeping the safety distance, stopping or parking when forbidden or dangerous, not indicating or wrongly indicating a manoeuvre, driving in the wrong direction, driving in a prohibited space, participating in unauthorised competitions or races**
**Unknown**	5315	43.1%	3312	44.2%	2003	41.5%	
**Speed**							
**No infraction**	6249	50.7%	3764	50.3%	2485	51.4%	**Adequate speed**
**Yes**	303	2.5%	248	3.3%	55	1.1%	**Inadequate speed for road conditions, exceeding the established speed or going too slowly/hindering circulation**
**Unknown**	5766	46.8%	3476	46.4%	2290	47.4%	
**Other infringement**							
**No infraction**	4730	38.4%	2865	38.3%	1865	38.6%	**No infractions**
**Yes**	201	1.6%	115	1.5%	86	1.8%	**Not using adequate lights, dazzling headlights, badly conditioned load, excess of load, load detachment, opening doors without precaution, excess of occupants, another infraction**
**Unknown**	7387	60.0%	4508	60.2%	2879	59.6%	
**Responsible**							
**No**	3374	27.4%	2345	31.3%	1029	21.3%	**The driver/rider is not responsible**
**Yes**	4359	35.4%	2181	29.1%	2178	45.1%	**The driver/rider is responsible**
**Unknown**	4585	37.2%	2962	39.6%	1623	33.6%	
**Distraction**							
**No**	3071	24.9%	1951	26.1%	1120	23.2%	**No distracting factors**
**Yes**	377	3.1%	214	2.9%	163	3.4%	**Use of mobile phone, use of hand-free devices, use of GPS devices, radio or music on, watching DVD or video device, wearing headphones, smoking, simultaneous driving activities (eating, drinking, finding objects…), interacting with other occupants, distracted by a previous collision, looking at the environment (landscape, advertising, signs...), lost in thought or absent minded, sleep, fatigue, sudden illness, indisposition**
**Unknown**	8870	72.0%	5323	71.1%	3547	73.4%	
**Errors**							
**No**	3303	26.8%	2275	30.4%	1028	21.3%	**No errors**
**Yes**	2044	16.6%	960	12.8%	1084	22.4%	**Failing to see a road sign, failing to see a vehicle/pedestrian/obstacle, not understanding a road sign or confused by it, hesitation or delay in making a decision, incorrect execution of a manoeuvre or inadequate manoeuvre, forgetting to signalise (with the vehicle indicators or lights…)**
**Unknown**	6971	56.6%	4253	56.8%	2718	56.3%	
**Seriously injured or killed?**							
**No**	11521	93.5%	6711	89.6%	4810	99.6%	**Moderately injured or uninjured in the collision**
**Yes**	797	6.5%	777	10.4%	20	0.4%	**Seriously injured or killed in the collision**
**Incident involving one or more motor vehicles?**							
**Yes**	9678	78.6%	4880	65.2%	4798	99.3%	**Motor vehicles involved in the collision**
**No**	2608	21.2%	2608	34.8%	0	0.0%	**No motor vehicles involved in the collision**

**Table 2:** Injured cyclist collisions.

Type of Injury	Drivers or Riders Involved In a Collision in Which One or More Cyclists were Injured	Cyclists	Other Road Users (Motorists)
**Uninjured**	4884	258 (3.4%)	4626 (95.8%)
**Slightly injured**	6637	6453 (86.2%)	184 (3.8%)
**Seriously injured**	728	711 (9.5%)	17 (0.4%)
**Killed**	69	66 (0.9%)	3 (0.1%)
**Total**		7488	4830

**Table 3:** Probability of cyclist KSI risk associated with different general variables.

Factor	Variables	KSI
**Other vehicle involvement**	**No motor vehicles involved**	0.120
	**Car**	0.101
	**Motorcycle**	0.081
	**Truck**	0.239
	**Bus**	0.133
	**Others**	0.238
**Bicycle infrastructure present**	**Yes**	0.088
	**No**	0.122
**Zone**	**Urban**	0.079
	**Inter-urban or urban highway**	0.190
**Traffic calming (30 kph or less)**	**Yes**	0.083
	**No**	0.119
**Intersection**	**Yes**	0.110
	**No**	0.111
**Weather**	**Clear**	0.116
	**Other**	0.113
**Surface**	**Good**	0.113
	**Other**	0.114
**Light**	**Good**	0.114
	**Other**	0.095
**Visibility**	**Good**	0.203
	**Other**	0.270

**Table 4:** Probability of cyclists KSI. Effect of variables related to cyclists and to other drivers/riders.

Variables	Value	KSI
**Age**		
**Cyclist**	<18	0.093
	18–25	0.094
	25–40	0.115
	40–60	0.128
	>60	0.091
**Motorist**	<18	0.160
	18–25	0.107
	25–40	0.108
	40–60	0.122
	>60	0.089
**Gender**		
**Cyclist**	Men	0.113
	Women	0.101
**Motorist**	Men	0.111
	Women	0.108
**Seat-belt**		
**Motorist**	Yes	0.109
	No	0.118
**Driving-Licence**		
**Motorist**	Yes	0.113
	No	0.112
**Helmet**		
**Cyclist**	Yes	0.142
	None	0.108
**Motorist**	Yes	0.136
	None	0.161
**Infringement**		
**Cyclist**	no infraction	0.115
	Yes	0.112
**Motorist**	no infraction	0.114
	Yes	0.113
**Speed**		
**Cyclist**	no infraction	0.115
	speed infraction	0.116
**Motorist**	no infraction	0.114
	speed infraction	0.113
**Other Infringement**		
**Cyclist**	no infraction	0.123
	Yes	0.097
**Motorist**	no infraction	0.119
	Yes	0.106
**Responsible**		
**Cyclist**	no	0.116
	Yes	0.109
**Motorist**	no	0.111
	Yes	0.112
**Distraction**		
**Cyclist**	no	0.119
	Yes	0.126
**Motorist**	no	0.119
	Yes	0.123
**Errors**		
**Cyclist**	no	0.118
	Yes	0.123
**Motorist**	no	0.117
	Yes	0.120

**Table 5:** Probability of collision locations. An analysis by different vehicle types.

Bicycle Infrastructure	Yes	No
**Bicycle**	0.057	0.447
**Car**	0.022	0.513
**Motorcycle**	0.052	0.447
**Truck**	0.018	0.569
**Bus**	0.023	0.486
**Others**	0.024	0.571
**Urban Zone**	**Urban Zone**	**Non-Urban (or major)**
**Bicycle**	0.693	0.307
**Car**	0.754	0.246
**Motorcycle**	0.836	0.164
**Truck**	0.466	0.534
**Bus**	0.756	0.244
**Others**	0.553	0.447
**‘30 zone’**	**Yes**	**No**
**Bicycle**	0.093	0.520
**Car**	0.108	0.503
**Motorcycle**	0.110	0.530
**Truck**	0.070	0.577
**Bus**	0.105	0.537
**Others**	0.080	0.614

**Table 6:** KSI risk by place, for different involved vehicle.

KSI	Urban	Non-Urban
**Car**	0.077	0.176
**Motorcycle**	0.055	0.211
**Truck**	0.141	0.325
**Bus**	0.077	0.308
**Others**	0.200	0.286
**KSI**	**Zone 30**	**Others**
**Car**	0.079	0.110
**Motorcycle**	0.058	0.093
**Truck**	0.156	0.258
**Bus**	0.083	0.149
**Others**	0.206	0.248

**Table 7:** KSI risk by involvement or not of motor vehicles, and presence of cycling infrastructure.

KSI	Bicycle Infrastructure	No Bicycle Infrastructure
**Collision involving motor vehicles**	0.086	0.118
**Collision not involving motor vehicles**	0.096	0.136
